# Multidisciplinary approaches to combat emerging viruses: diagnostics, therapeutic gene and vaccine delivery, and nanotherapeutics

**DOI:** 10.3389/fmicb.2024.1387623

**Published:** 2024-06-20

**Authors:** Xianqiang Yu, Qing He, Qingming Kong

**Affiliations:** ^1^Medical College of Qingdao University, Qingdao, China; ^2^School of Basic Medicine and Forensics, Key Laboratory of Bio-tech Vaccine of Zhejiang Province, Engineering Research Center of Novel Vaccine of Zhejiang Province, Hangzhou Medical College, Hangzhou, China; ^3^Key Laboratory of Biomarkers and In Vitro Diagnosis Translation of Zhejiang province, School of Laboratory Medicine and Bioengineering, Hangzhou Medical College, Hangzhou, China

**Keywords:** virus, CRISPR, vaccine, mRNA, monoclonal antibodies

## Abstract

Emerging viruses, such as filoviruses (Ebola, Marburg), SARS and MERS coronaviruses, and Zika, pose significant threats to global public health, particularly for individuals with co-morbidities. To address these challenges, this review article explores multidisciplinary strategies for combatting emerging viruses. We emphasize the importance of developing accurate diagnostics, innovative therapeutic gene and vaccine delivery systems, and long-acting nanotherapeutics. These approaches are designed to enhance the safety and efficacy of treatments against these deadly pathogens. We discuss the collaborative efforts of virologists, geneticists, formulation scientists, clinicians, immunologists, and medicinal chemists in advancing these therapeutic modalities.

## 1 Introduction

Emerging viruses, such as filoviruses (Ebola, Marburg), SARS and MERS coronaviruses, and Zika, continue to pose significant threats to global public health, particularly for vulnerable populations with co-morbidities (Mehedi et al., 2011; Zhang et al., 2014; Murray, 2015; Gharbaran and Somenarain, 2019; Li and Du, 2019; Nicastri et al., 2019; Dieterle et al., 2020; Dong et al., 2020). Addressing these threats demands a comprehensive strategy that encompasses the development of precise diagnostics and the pursuit of safe and effective therapeutic modalities (Morse et al., 2020; Majumder and Minko, 2021). Accurate and highly sensitive diagnostics are the cornerstone of effective disease management. Early detection is essential for timely intervention and containment. In parallel, the development of novel therapeutic modalities is crucial to address the challenges posed by emerging viruses (Fauci and Morens, 2012; Anema et al., 2014; McCloskey et al., 2014; Hui and Zumla, 2015). This review will delve into the latest advances in diagnostic techniques that enable rapid identification and surveillance of viral outbreaks. Additionally, we will explore the innovative therapeutic strategies that hold the potential to restrict and ultimately eliminate these viruses. From mRNA vaccines and RNA/CRISPR modalities to long-acting nanotherapeutics, these emerging solutions aim to enhance the safety and efficacy of treatments for patients, especially those with co-morbidities (Kushnir et al., 2012; Hilton et al., 2015; Al-Halifa et al., 2019; Maruggi et al., 2019; Van Hoecke and Roose, 2019; Burmistrz et al., 2020; Abudayyeh and Gootenberg, 2021).

Realizing these goals necessitates a collaborative effort that transcends traditional boundaries between scientific disciplines. Virologists, geneticists, formulation scientists, clinicians, immunologists, and medicinal chemists must join forces to drive these innovations from the laboratory to clinical studies. This special issue serves as a vital platform for the exchange of valuable information encompassing both basic and applied research. By uniting the collective expertise of these diverse fields, we can accelerate the development of diagnostics, mRNA vaccines, RNA/CRISPR modalities, and long-acting nanotherapeutics (McCloskey et al., 2014; Hilton et al., 2015). Together, we aim to forge a path toward more efficient and safer treatments against emerging viruses, thereby safeguarding the health and well-being of individuals, particularly those most vulnerable to these formidable pathogens.

## 2 Diagnostics for early detection

Early detection and accurate diagnosis play pivotal roles in the effective management and containment of emerging viral infections, particularly in individuals with co-morbidities (Table 1) (Anema et al., 2014). Rapid and precise identification of the causative agents is crucial for timely intervention, implementation of public health measures, and initiation of appropriate therapeutic strategies.

**Table 1:** Different diagnostic technologies for early detection of emerging viral infections based on accuracy, ease of use, and efficiency.

Diagnostic technology	Accuracy	Ease of use	Efficiency
Polymerase Chain Reaction (PCR) assays	High sensitivity and specificity for detecting viral nucleic acids	Requires trained personnel and specialized equipment; longer turnaround time compared to some rapid tests	Time-consuming but highly accurate; suitable for laboratory settings with proper infrastructure
Next-generation sequencing (NGS)	Comprehensive genomic profiling; aids in identifying novel viral strains and their evolutionary patterns	Complex data analysis; requires specialized equipment and expertise	Time-consuming; useful for research and surveillance purposes to understand viral evolution and transmission
Serological assays (e.g., ELISA)	Detects viral antigens or antibodies; useful for acute and convalescent phase diagnosis	Relatively simple procedure; can be automated for high throughput	Moderate to high turnaround time; may require additional confirmatory tests for accurate diagnosis
Rapid point-of-care testing devices	Varies depending on the technology used; generally lower sensitivity compared to PCR assays	Simple operation; suitable for use in resource-limited settings or at the point of care	Rapid results within minutes to hours; may sacrifice sensitivity for speed
Microfluidic technologies and lab-on-a-chip devices	Varies depending on the application; can offer high sensitivity and specificity	Compact and portable; suitable for on-site testing and point-of-care diagnostics	Rapid results; minimal sample volume required; may require specialized training for operation
Biosensors	Highly sensitive and specific detection platforms; customizable for different viral targets	Variable depending on the design; may require specialized knowledge for development and optimization	Rapid results with potential for real-time data analysis; may require calibration and validation

Cutting-edge diagnostic technologies have revolutionized our ability to swiftly identify and characterize emerging viruses. Polymerase chain reaction (PCR) assays, including real-time PCR, offer high sensitivity and specificity for detecting viral nucleic acids (Blyn et al., 2008; Ma and Khan, 2009). Next-generation sequencing (NGS) allows for comprehensive genomic profiling, aiding in the identification of novel viral strains and their evolutionary patterns (Voelkerding et al., 2009; Lee et al., 2017). Moreover, serological assays, such as enzyme-linked immunosorbent assays (ELISA), enable the detection of viral antigens or antibodies, contributing to both acute and convalescent phase diagnosis (Johnson et al., 2000).

The development of rapid and portable point-of-care testing devices has significantly enhanced our capacity for on-site diagnostics. These devices facilitate prompt detection in resource-limited settings, enabling healthcare providers to quickly assess and respond to potential outbreaks (Kumar et al., 2021). The integration of microfluidic technologies and lab-on-a-chip devices further streamlines the diagnostic process, allowing for efficient sample processing and analysis.

Biosensors, leveraging nanotechnology, provide highly sensitive and specific detection platforms (Xu et al., 2016; Lai et al., 2018). Nanoparticles functionalized with viral-specific ligands can selectively bind to viral components, leading to measurable signals. This technology not only enhances detection sensitivity but also enables the development of compact and portable diagnostic devices. Additionally, the use of biosensors in conjunction with artificial intelligence algorithms holds promise for real-time data analysis, further expediting diagnosis. These platforms utilize various mechanisms to detect specific pathogens, often through the interaction between viral components and functionalized nanoparticles or surfaces. For instance, in the case of detecting viruses such as Zika, biosensors may utilize nanoparticles functionalized with viral-specific ligands (Vorou, 2016). These ligands can selectively bind to viral components, leading to measurable signals that indicate the presence of the virus. Specificity and sensitivity are crucial aspects of biosensors, ensuring accurate detection of the target pathogen while minimizing false positives and negatives. Researchers often validate biosensors using known samples of the target virus and comparing the results with established diagnostic methods. Sensitivity refers to the ability of the biosensor to detect low concentrations of the virus, while specificity refers to its ability to distinguish the target virus from other substances present in the sample.

Continuous monitoring and surveillance are critical components of early detection. Integration of epidemiological data, environmental monitoring, and sentinel surveillance systems enables the identification of unusual patterns or spikes in disease incidence (Korber et al., 2020; Rambaut et al., 2020). Early warning systems, supported by data analytics and machine learning, empower public health authorities to respond proactively to potential outbreaks, mitigating the impact of emerging viruses on vulnerable populations.

In summary, early detection and diagnosis of emerging viral infections demand a synergistic approach that combines traditional and innovative diagnostic modalities. The integration of advanced technologies, point-of-care testing, nanotechnology, and robust surveillance systems collectively contribute to a comprehensive strategy for swift and accurate identification of viral threats, forming the first line of defense in protecting individuals, especially those with co-morbidities, from the impacts of these formidable pathogens.

## 3 Therapeutic gene and vaccine delivery

The development of effective therapeutic gene and vaccine delivery systems is imperative in the fight against emerging viral infections, especially in individuals with co-morbidities. Harnessing innovative technologies, such as mRNA vaccines, RNA/CRISPR modalities, and long-acting nanotherapeutics, presents promising avenues for enhancing treatment safety and efficacy.

mRNA vaccines represent a groundbreaking approach in vaccinology (Maruggi et al., 2019). They leverage synthetic messenger RNA to instruct cells to produce viral antigens, eliciting robust immune responses. For instance, in the case of SARS-CoV-2, mRNA vaccines have played a pivotal role in responding to evolving viral threats. Similarly, RNA interference (RNAi) and CRISPR Cas9 systems offer powerful tools for targeted intervention against emerging viruses (Hilton et al., 2015). RNAi can inhibit viral replication by silencing essential viral genes, while CRISPR Cas9 enables precise editing of viral genomes, disrupting key viral functions.

New CAS9 enzyme variants have been developed to target both DNA and RNA viruses. For DNA viruses, variants of the CRISPR-Cas9 system such as SpCas9-NG and SpCas9-NG3 have been engineered to enhance specificity and activity. These variants have been optimized to recognize target sequences with greater precision, reducing off-target effects and improving the efficiency of viral genome editing (Chen et al., 2017). In the case of RNA viruses, recent advancements have led to the development of RNA-targeting CRISPR-Cas systems. For instance, the Cas13 family of enzymes, particularly Cas13a (previously known as C2c2), has been repurposed for RNA targeting. Cas13a exhibits RNA-guided RNase activity, allowing for specific cleavage of viral RNA molecules. This capability makes Cas13a an attractive candidate for targeting RNA viruses, including SARS-CoV-2 and influenza (Abudayyeh et al., 2017). Additionally, engineered variants of Cas13, such as Cas13b and Cas13d, have been developed to further improve RNA targeting specificity and efficiency. These variants offer expanded targeting capabilities and enhanced sensitivity, enabling precise manipulation of viral RNA sequences for therapeutic purposes (Konermann et al., 2018).

Nanotherapeutics, particularly long-acting formulations, hold promise for sustained drug release and improved patient compliance (Kushnir et al., 2012; Al-Halifa et al., 2019). These nanoparticles can encapsulate therapeutic agents, including genes, inhibitors, and biologics, providing controlled release and reducing administration frequency. However, it’s important to note that nanotherapeutics may encounter challenges related to biocompatibility and clearance, which need to be carefully addressed. Nanoparticles have emerged as versatile carriers for therapeutic agents, facilitating targeted and sustained release. Lipid nanoparticles have been extensively utilized as delivery vehicles for nucleic acid-based therapeutics, including mRNA vaccines. For example, the Pfizer-BioNTech and Moderna COVID-19 vaccines utilize LNPs to encapsulate and deliver mRNA encoding the spike protein of SARS-CoV-2, eliciting an immune response against the virus (Polack et al., 2020; Baden et al., 2021). Lipid nanoparticles have also been investigated for the delivery of small interfering RNA (siRNA) targeting viral genes. By encapsulating siRNA within LNPs, researchers have demonstrated effective inhibition of viral replication in preclinical models of respiratory syncytial virus (RSV), influenza, and hepatitis B virus (HBV) infections (van Rijt et al., 2014; Dong et al., 2015). In the context of emerging viral infections, long-acting nanotherapeutics offer several advantages. These nanoparticles can encapsulate antiviral agents, including therapeutic genes, small molecule inhibitors, and biologics, providing a controlled and prolonged release. This approach not only enhances the therapeutic efficacy but also reduces the frequency of administration, improving patient compliance and overall treatment outcomes. Although nanoparticles offer prolonged release of vaccines/drugs, they may also trigger immune responses, potentially leading to adverse reactions. To mitigate these issues, researchers employ various strategies to modulate the interaction between nanoparticles and the immune system. One approach is surface modification of nanoparticles to minimize immune recognition and activation. Coating nanoparticles with polymers or modifying their surface properties can reduce interactions with immune cells and mitigate inflammatory responses. Additionally, optimizing the size, shape, and composition of nanoparticles can influence their biodistribution and immunogenicity, thereby minimizing immune system triggering.

While innovative technologies like mRNA vaccines, RNA/CRISPR modalities, and long-acting nanotherapeutics hold great promise in combating emerging viral infections, they also face challenges and limitations that need to be addressed. One major challenge with mRNA vaccines is their requirement for ultra-cold storage conditions, which can pose logistical challenges, especially in resource-limited settings. Additionally, mRNA vaccines may induce adverse reactions in some individuals, although these are typically mild and transient. To overcome these limitations, efforts are underway to develop mRNA vaccine formulations that are more stable at higher temperatures and to enhance our understanding of vaccine safety profiles through ongoing surveillance and research. While RNAi and CRISPR Cas9 systems offer powerful tools for targeted intervention against emerging viruses, they may encounter off-target effects, potentially leading to unintended consequences. Researchers are actively working to improve the specificity and efficiency of these modalities through optimization of delivery methods, sequence design, and Cas9 variants. Additionally, careful screening and validation procedures are essential to minimize off-target effects and ensure the safety and efficacy of RNA/CRISPR-based therapies.

In conclusion, therapeutic gene delivery and vaccine development represent dynamic frontiers in the battle against emerging viral infections. This Special Issue serves as a platform to explore and share the latest advancements in these areas, fostering collaboration among experts from diverse disciplines to accelerate the development of safe and effective treatments for individuals, especially those with co-morbidities, facing the threats of Ebola, Marburg, SARS, MERS, Zika, and other emerging viruses.

## 4 Nanotherapeutics for prolonged action

Long-acting nanotherapeutics have gained prominence for their ability to provide sustained release of antiviral agents. These systems offer the advantage of prolonged drug exposure, reducing the frequency of administration and enhancing patient compliance. The development of long-acting nanotherapeutics represents a promising frontier in the battle against emerging viral infections, offering innovative solutions to enhance the safety and efficacy of treatments, especially for individuals with co-morbidities.

Lipid and polymeric nanoparticulate systems serve as versatile platforms for drug delivery, providing a protective and controlled environment for encapsulated therapeutic agents (Kushnir et al., 2012; Al-Halifa et al., 2019). In the context of emerging viral infections, these nanoparticles offer several advantages. Lipid nanoparticles, for instance, can encapsulate antiviral drugs, siRNAs, or mRNA vaccines, facilitating their targeted delivery to specific cells or tissues. Polymeric nanoparticles, on the other hand, enable sustained release, prolonging the therapeutic effect and reducing the need for frequent administration.

The use of nanotherapeutics extends beyond conventional antiviral drugs to include biologics and small molecule inhibitors. By encapsulating monoclonal antibodies, fusion proteins, or peptides within nanoparticles, the stability and bioavailability of these therapeutic agents are improved (Chakravarty and Vora, 2020). This approach not only extends their half-life but also allows for controlled release, optimizing the antiviral response and minimizing potential side effects. Long-acting nanotherapeutics aim to provide sustained drug exposure over an extended period, addressing the challenge of maintaining therapeutic levels in the body (Al-Halifa et al., 2019). This sustained release not only enhances the efficacy of antiviral treatments but also contributes to patient compliance by reducing the frequency of administration. For individuals with co-morbidities, this can be particularly advantageous, minimizing the burden of frequent medical interventions. Nanoparticulate systems can be designed for precision targeting, directing therapeutic agents specifically to infected cells or tissues. This targeted delivery not only increases the concentration of the antiviral agent at the site of infection but also minimizes off-target effects, improving overall safety. Moreover, the enhanced bioavailability of encapsulated agents contributes to the therapeutic effectiveness of nanotherapeutics against emerging viruses.

## 5 Targeted therapies

Addressing the threats posed by emerging viruses necessitates the development of precise and targeted therapeutic interventions. The advent of targeted therapies offers a promising approach to restrict and eliminate viruses, particularly in individuals with co-morbidities.

Monoclonal antibodies (mAbs) are engineered to specifically recognize and bind to viral antigens, preventing their interaction with host cells and neutralizing the virus (Breedveld, 2000; Brennan et al., 2010; Taylor et al., 2021). In the context of emerging viral infections, mAbs can be designed to target key viral proteins, disrupting viral entry, replication, or assembly. The specificity of mAbs allows for precision in targeting the virus while minimizing impact on host cells. Collaborative efforts among immunologists, geneticists, and clinicians are crucial for identifying optimal antibody targets and designing effective therapeutic regimens.

Fusion proteins are engineered molecules that combine the functional domains of different proteins to create a single therapeutic agent with enhanced efficacy (Apellániz et al., 2014). In the context of emerging viruses, fusion proteins can be designed to target specific steps in the viral life cycle. For instance, a fusion protein might combine elements that inhibit viral entry and replication simultaneously (White et al., 2008). This strategy leverages the synergistic effects of multiple antiviral mechanisms within a single therapeutic entity.

Antiviral peptides represent a class of targeted therapies that can disrupt viral processes through their ability to interact with viral proteins or cellular receptors (Wang et al., 2010; Jesus et al., 2012). Peptides can inhibit viral fusion, entry, or replication by interfering with key interactions. The development of antiviral peptides requires collaboration between medicinal chemists, virologists, and formulation scientists to optimize peptide design, stability, and delivery for effective therapeutic outcomes.

In addition, CRISPR Cas9 technology offers a revolutionary approach for targeted genome editing. In the context of emerging viral infections, CRISPR Cas9 systems can be designed to specifically target and modify viral genomes, disrupting essential viral genes and inhibiting replication (Hilton et al., 2015).

## 6 The role of multidisciplinary collaboration

Successful development and implementation of these emerging modalities rely on collaboration among virologists, geneticists, formulation scientists, clinicians, immunologists, medicinal chemists, and other experts. We emphasize the importance of interdisciplinary teamwork in achieving the ultimate goal of efficient and safe treatments against emerging viruses.

Virologists play a pivotal role in understanding the intricacies of viral replication, transmission, and pathogenesis. Collaborating with geneticists enables the identification of viral targets for therapeutic interventions, including the design of diagnostics and the development of mRNA vaccines, RNA/CRISPR modalities, and targeted antiviral therapies. The insights from both fields contribute to a comprehensive understanding of the viral lifecycle and aid in the identification of vulnerabilities for targeted interventions.

Clinicians play a critical role in translating laboratory findings into real-world patient care. Their insights into the clinical manifestations of viral infections, patient demographics, and treatment outcomes are invaluable for guiding research priorities. Collaboration with virologists and formulation scientists helps bridge the gap between bench and bedside, ensuring that emerging diagnostics and therapeutic modalities are not only effective but also feasible for clinical implementation.

Immunologists contribute their expertise in understanding host immune responses to viral infections. Collaborating with virologists, they help identify potential targets for immunotherapeutic modalities, such as monoclonal antibodies and fusion proteins. The interaction between immunologists and geneticists is crucial for designing vaccines that elicit robust and targeted immune responses, contributing to the development of mRNA vaccines and RNA/CRISPR modalities.

It needs to be stressed that combining different modalities indeed holds the potential to enhance therapeutic outcomes and broaden the spectrum of treatment options for emerging viral infections. By leveraging the complementary mechanisms of action of multiple modalities, researchers can achieve synergistic effects that enhance antiviral efficacy and reduce the likelihood of resistance development. For example, combining mRNA vaccines with long-acting nanotherapeutics can prolong the duration of immune stimulation, leading to more robust and durable immune responses. Similarly, combining RNA/CRISPR modalities with monoclonal antibodies or antiviral peptides can provide dual mechanisms of action against the virus, enhancing therapeutic efficacy and reducing the risk of treatment failure.

## 7 Conclusion

In the face of unprecedented challenges posed by emerging viruses such as filoviruses, SARS and MERS coronaviruses, and Zika, the imperative for innovative and collaborative solutions has never been more apparent. This review has underscored the critical importance of multidisciplinary approaches in developing effective strategies against these viral threats. By bringing together the expertise of virologists, geneticists, formulation scientists, clinicians, immunologists, and medicinal chemists, we have explored a diverse array of advancements in diagnostics, therapeutic gene and vaccine delivery, and nanotherapeutics.

The journey toward efficient and safe treatments begins with accurate and highly sensitive diagnostics, enabling early detection and response. The collaborative efforts of virologists, geneticists, and clinicians in this realm pave the way for timely interventions, essential for the containment of emerging viral outbreaks. On the therapeutic front, the exploration of long-acting prodrugs, nanoparticles, immunotherapeutic modalities, and CRISPR Cas9 systems has illuminated promising avenues for viral restriction and eventual elimination. The collaboration among immunologists, geneticists, formulation scientists, and medicinal chemists has been instrumental in the design and optimization of targeted therapies. Monoclonal antibodies, fusion proteins, peptides, and innovative gene-editing technologies hold the potential to revolutionize the landscape of antiviral treatments.

The vision set forth by this Special Issue revolves around the creation of a robust multidisciplinary platform. This collaborative space fosters the interchange of valuable information, bridging the gap between basic and applied research. The synergy arising from the collective knowledge and skills of diverse experts propels the development of diagnostics, mRNA vaccines, RNA/CRISPR modalities, and long-acting nanotherapeutics.

As we navigate the complexities of combatting emerging viruses, the collaborative spirit embodied in this review acts as a guiding beacon. By transcending traditional boundaries and working in tandem, scientists and clinicians from various disciplines contribute to a unified front against these formidable pathogens. The ongoing and future endeavors of this multidisciplinary community are essential for turning the tide in the battle against emerging viral infections and ensuring the efficient and safe treatment of individuals, particularly those with co-morbidities, on a global scale.

## Author contributions

XY: Methodology, Investigation, Formal analysis, Writing – original draft. QH: Writing – original draft. QK: Funding acquisition, Project administration, Supervision, Writing – review & editing.
